# COVID-19 and Unmet Healthcare Needs of Older People: Did Inequity Arise in Europe?

**DOI:** 10.3390/ijerph18179177

**Published:** 2021-08-31

**Authors:** Marta González-Touya, Alexandrina Stoyanova, Rosa M. Urbanos-Garrido

**Affiliations:** 1 Servicio de Medicina Preventiva y Salud Pública, Hospital Universitario Ramón y Cajal, 28034 Madrid, Spain; mgtouya@salud.madrid.org; 2 Department of Economics, Faculty of Economics and Business, University of Barcelona, 08034 Barcelona, Spain; 3 Department of Applied Economics, Public Economics and Political Economy, Faculty of Economics and Business, University Complutense of Madrid, 28223 Madrid, Spain; urbanos@ccee.ucm.es

**Keywords:** unmet needs, COVID-19, SHARE survey, inequalities, horizontal inequity

## Abstract

Background: The disruption in healthcare provision due to the COVID-19 pandemic forced many non-urgent medical treatments and appointments to be postponed or denied, which is expected to have huge impact on non-acute health conditions, especially in vulnerable populations such as older people. Attention should be paid to equity issues related to unmet needs during the pandemic. Methods: We calculated concentration indices to identify income-related inequalities and horizontal inequity in unmet needs due to postponed and denied healthcare in people over 50 during COVID-19, using data from the Survey on Health, Ageing and Retirement in Europe (SHARE). Results: Very few countries show significant income-related inequalities in postponed, rescheduled or denied treatments and medical appointments, usually favouring the rich. Only Estonia, Italy and Romania show a significant horizontal inequity (HI) in postponed healthcare, which apparently favours the poor. Significant pro-rich inequity in denied healthcare is found in Italy, Poland and Greece. Conclusions: Although important income-related horizontal inequity in unmet needs of European older adults during the early waves of the COVID-19 pandemic is not evident for most countries, some of them have to carefully monitor barriers to healthcare access. Delays in diagnosis and treatments may ultimately translate into adverse health outcomes, reduced quality of life and, even, widen socio-economic health inequalities among older people.

## 1. Introduction

Equity in access to health care has been set as a central objective of most health care systems. This implies that services should be accessible on the basis of need rather than on specific socio-economic circumstances (e.g., ability to pay or geographical location), race or sex. Ensuring equal access to health care is considered a major social determinant of health improvements and a tool for addressing health inequalities by social status [1]. Although universal health coverage characterises most European health systems, a basic feature to ensure equal access for equal need, many countries still face important access barriers. These may have intensified during the COVID-19 pandemic, when the priority of the health sector was infection prevention and control.

The COVID-19 pandemic forced health care systems all around the world to reorganize most of their services to adapt to many unprecedented circumstances, to cope with uncertainty and, very probably, to leave aside equity concerns [2]. Even though some European countries extended entitlement to publicly financed health services to everyone, regardless of residence or insurance status (such as Portugal or France, among others), or suspended user charges for some services (as in Ireland or Belgium) [3], new barriers to healthcare access arose.

The high viral transmission and its capability to induce extremely severe cases led to a rapid overload of most facilities which urged both health managers and health professionals to prioritize the assistance of COVID-19 cases over any other scheduled visits or procedures. Many elective surgeries and non-urgent treatments had to be postponed to free up human resources and hospital beds and the delivery of outpatient care had to be significantly altered [4]. Also, a part of the demand for health care could not be met in healthcare facilities and was provided remotely or even remained unmet. Moynihan et al. [5] reviewed the health system response to COVID-19 in 20 countries and estimated that healthcare utilization decreased by more than one third during the pandemic (37%). The drop in services overall was comprised of reductions in visits (42%), admissions (28%), diagnostics (31%), and therapeutics (30%). These data suggest that access to diagnostic services and medical advice was seriously hampered to prevent the COVID-19 spread and to reduce morbidity and mortality in the outbreak of the pandemic. As older adults are more susceptible to get infected and are at higher risk of developing a severe disease and serious complications, they have been particularly affected by the new access barriers.

This disruption in health care provision is thought to have had a great impact on acute non-COVID-19 conditions assistance, on regular care of chronic illnesses as well as on early diagnosis and treatment of life-threatening diseases [5,6,7,8,9,10]. For instance, cancer services had to adapt to the new situation: chemotherapy protocols were revised, maintenance therapy for some tumours was cancelled and the participation in clinical trials and experimental therapies was suspended [6]. There is also evidence suggesting a significant reduction in systemic anticancer treatment initiation during the first months of the pandemic [10]. In addition, the COVID-19 outbreak and the subsequent restrictions are shown to result in a significant drop in primary care contacts for almost all conditions, and in particular, for diabetic emergencies, depression and self-harm [11]. Older adults are found to be disproportionately affected by delays in cancer diagnosis and treatment [12], and by significant reductions in the number of physician consultations, specialist referrals and hospital admissions [13]. 

This might have resulted in serious adverse health consequences, as unmet health care needs have been associated with an increase in mortality rates [9,14]. Maringe et al. [9] estimated that the total additional years of life lost in the UK as a result of diagnostic delays in avoidable cancer conditions caused by the COVID-19 pandemic could exceed 60,000 years. Existing previous research linking unmet needs and mortality during the 2008 economic crisis [14] gives a hint on the potential health impacts beyond the COVID-19 pandemic. Lindström et al. [14] demonstrated that unmet healthcare needs were significantly associated with increased mortality for all causes, except for cardiovascular disease [14]. Furthermore, adverse health consequences seem to be particularly severe for the most vulnerable population groups, such as older and frail patients [11,15] or lower-income individuals [16], for whom chronic non-communicable diseases and multimorbidity are more prevalent. 

The overall impact on general health indicators will likely be particularly important in Europe, since the proportion of older adults in the European countries is the highest in the world. In 2019, people aged 55 or over represented one third (33.6%) of the total EU-27 population and it was higher in 10 countries, reaching 36.5% in Italy [17].

The aim of this paper is to identify if there has been any income-related discrimination in unmet healthcare needs of the European older people during the first months of the pandemic and, if that is the case, to quantify its magnitude. Our final goal is to unravel if the response to the non-COVID problems may have contributed to enlarge the existing socio-economic inequalities in health. To do that we use the COVID-19 questionnaire of the Survey of Health, Ageing and Retirement in Europe (SHARE) and focus on different dimensions of unmet need that may represent access barriers linked to the provision of health care [18,19]. In particular, we analyse information about postponement or rescheduling by a health professional of medical appointments due to COVID-19, and also about denied medical appointments during the pandemic. Although the SHARE also provides information about forgoing care during the outbreak of COVID-19 due to fear of getting infected, we exclude this variable since it represents a dimension of unmet need mainly attributed to demand, as it is linked to patients’ perception of danger and their consequent choice of action.

Several studies have tried to assess the degree of equity in the use of long-term care and health care among European older adults in a pre-pandemic context [20,21,22,23,24]. Rodrigues et al. [20] observed pro-poor inequity in informal care for many European countries, but also showed inequity favouring the rich in the delivery of home care for Italy and Spain. This latter result is confirmed by Ilinca et al. [21]. García-Gómez et al. [22] also found pro-rich inequity in home care for disabled Spaniards, but pro-poor inequity in informal care and unmet needs for long-term care services. More recently, Lera et al. [23] reported pro-rich inequity in both formal and informal long-term care for a wide sample of European countries. Furthermore, Tavares and Zantomio [24] estimated significant pro-rich inequity in the use of specialists’ visits for southern European countries, and in primary care for Portugal.

However, the evidence on the evolution of income-related inequalities and equity in health care during the COVID-19 pandemic is still scarce, and only refers to the United Kingdom [25]. Furthermore, only a couple of studies analysed the determinants of unmet needs during the COVID-19 outbreak for European older people [26,27]. Unlike our paper, these studies neither provide information disaggregated by countries, nor calculate inequity in unmet need.

We contribute to the literature on equity in healthcare utilisation by analysing horizontal equity in the distribution of postponed and denied health care across Europe. To the best of our knowledge, this is the first paper that computes income-related inequalities and horizontal inequity in the distribution of unmet needs of the older adults during the COVID-19 pandemic across European countries. 

The paper is organized as follows: Section 2 presents the data and empirical strategy. The results are described in Section 3 and are discussed in Section 4. Section 5 concludes. 

## 2. Data and Methods

### 2.1. Data

Data come from the Survey of Health, Ageing and Retirement in Europe (SHARE), collected between June and August 2020 in 26 European countries and Israel [19]. Detailed information regarding survey participation rates and sampling procedure may be found in Scherpenzeel et al. [28]. The SHARE-COVID-19 wave was especially designed to explore the impact of the pandemic on a wide array of life domains [19]. It contains information on sociodemographic characteristics, health and health behaviours, use of health care services, social relationships, labour situation, housing arrangements, etc. We kept in the sample only individuals who gave valid responses to the questions regarding the unmet needs during the pandemic. In addition, to be able to assess income-related inequalities, we linked the 2020 data with baseline information from SHARE wave 7 [29]. The SHARE contains a very detailed information on households’ monetary earnings. These include earnings from employment or self-employment, old age pensions, retirement pensions, survivor and war pensions, private occupational pensions, disability pensions and benefits, sickness pensions and benefits, unemployment and insurance benefits, public care insurance benefits, as well as income from monetary and non-monetary assets. As in most surveys, the item non-response to the different questions regarding earnings is relatively high (around 20%). De Luca [30] deals with this problem by imputing income-related items and provides researchers with a household income variable which contains no missing values. The imputation procedure is explained in the SHARE Release Guide 7.0.0 [30] and follows De Luca et al. [31].

To guarantee a large enough number of observations allowing to perform cross-country comparisons, the income imputed values are used. We restricted the analysis to the European countries, excluding the Netherlands due to the high number of missing values on relevant variables. Our final sample consists of 41,606 non-institutionalised individuals aged 50 years or over.

### 2.2. Methods

We use concentration indices (CI) to measure inequality and inequity in the distribution of unmet needs, a method widely used in the health field [32,33,34]. The CI is based on the concentration curve, which represents the relationship between the cumulative proportion of population ranked by economic position (usually income) and the cumulative proportion of the variable of interest. The standard concentration index measures twice the area between the concentration curve and the 45° line and can be calculated by the following expression [32,35]:

(1) $$CI=\frac{2}{n^{2}\bar{y}}\sum_{i=1}^{n}y_{i}r_{i}$$

where $\bar{y}$ is the mean of the variable of interest, and $r_{i}$ is the cumulative percentage that each individual represents over the total population after being ranked. The CI ranges from −1 to +1. Negative (positive) values of the index mean that y tends to concentrate on the worse-off (better-off). When the CI is not significantly different from zero, there is no socio-economic inequality in the distribution of y.

One of the main advantages of the concentration indices is that they can be decomposed into their explanatory factors, which may include a vector of proxies for clinical need, but also non-need determinants [36]. Thus, inequity may be computed by subtracting from total inequality the need component, since need is considered as a legitimate source of variation in the demand for and utilization of health care. When the variable of interest links linearly and additively to a set of need (*x*) and non-need (*z*) variables, it may be written as:

(2) $$y_{i}=\alpha_{i}+\sum_{j}\beta_{j\;}x_{ji}+\sum_{k}\gamma_{k\;}z_{ki}+e_{i},$$

where *e* is the error term, and the CI may be expressed as a weighted sum of the partial CI for the explanatory factors of inequality, being the weight the elasticity of y with respect to $x_{j},\;z_{k}$:

(3) $$CI=\sum_{j}\frac{\left(\beta_{j}{\bar{x}}_{j}\right)}{\bar{y}}CI_{j}+\sum_{k}\frac{\left(\gamma_{k}{\bar{z}}_{k}\right)}{\bar{y}}CI_{k}+\frac{GCI_{e}}{\bar{y}}$$

where ${\bar{x}}_{j}$ and ${\bar{z}}_{k}$ are the mean of $x_{j},\;z_{k}$, respectively, and *GCI_e_* is the generalized concentration index for the error term. The horizontal inequity index (*HI*) may then be defined as the difference between the concentration index and the contribution of the need variables, as expressed by Equation (4):

(4) $$HI=CI-\sum_{j}\frac{\left(\beta_{j}{\bar{x}}_{j}\right)}{\bar{y}}CI_{j}$$

However, when y is represented by a binary variable, as it is our case, the CI ranges from $\bar{y}-1$ to $1-\bar{y}$ and some correction has to be done in order to compare indices across time or countries with significant differences in the means of y [37]. We employ the correction proposed by Erreygers [38]. Thus, the corrected concentration index (*CCI*) can be expressed by Equation (5):

(5) $$CCI=\frac{8}{n^{2}}\sum_{i=1}^{n}y_{i}r_{i}$$

Also, when the outcome variable is dichotomous, non-linear models (such as probit models) are preferred to estimate the variable of interest. Then, some linear approximation to the non-linear model has to be made in order to decompose the corrected concentration index. In this case, Equation (2) may be expressed by Equation (6):

(6) $$y_{i}=\alpha^{m}+\sum_{j}\beta_{j}^{m}x_{ji}+\sum_{k}\Upsilon_{k}^{m}z_{ki}+u_{i}$$

where $\alpha^{m}\;,\;$$\beta_{j\;}^{m}\;\mathrm{and}\;\;\gamma_{k}^{m}$ are the partial effects of the variables evaluated at sample means, and $u_{i}$ is the error term, including approximation errors. Consequently, Equations (3) and (4) are transformed, respectively, into Equations (7) and (8):

(7) $$CCI=\sum_{j}\frac{\left(\beta_{j}^{m}{\bar{x}}_{j}\right)}{\bar{y}}CCI_{j}+\sum_{k}\frac{\left(\gamma_{k}^{m}{\bar{z}}_{k}\right)}{\bar{y}}CCI_{k}+4\cdot GCI_{u}$$

(8) $$HI=CCI-\sum_{j}\frac{\left(\beta_{j}{\bar{x}}_{j}\right)}{\bar{y}}CCI_{j}$$

### 2.3. Definition of Variables

Unmet needs are proxied by two different variables. First, a variable indicating if there has been (or has not been) a postponed or rescheduled (by a health professional) medical appointment due to COVID-19. Second, a variable indicating if any medical appointment has been (or has not been) denied since the outbreak of the pandemic. Each variable is then represented by a dummy that takes the value of 1 if the individual reports unmet need, and 0 otherwise. The exact wording of the questions referred to unmet needs in the COVID-19 questionnaire is the following: Did you have a medical appointment scheduled, which the doctor or medical facility decided to postpone due to Corona? and Did you ask for an appointment for a medical treatment since the outbreak of Corona and did not get one? with only two possible answers (yes/no). Thus, it is impossible to distinguish between individuals who neither had a scheduled medical appointment postponed nor were denied an appointment and those who had no appointment at all or didn’t ask for any.

Moreover, the ranking variable is measured by equivalent household net income. Since the information regarding income collected in wave 8 had not been released yet, we use the most recent data available, corresponding to wave 7. We consider that this is a good proxy for present income, as significant variations in this variable are not expected for a major part of the sample (i.e., the retired people). The Buhmann equivalence scale is used to calculate equivalent income, since information on the age of the members of the household is not available. 

The remaining variables used in the analysis can be classified as need and non-need variables. Following previous literature, we classified age, gender and health status as need variables [22,24,39,40]. To account for age, we constructed six age-groups (50–64, 65–69, 70–74, 75–79, 80–84, and 85 and over). Gender is captured by a dummy variable that equals one for women and zero for men. The SHARE questionnaire contains a rich set of variables describing individuals’ health status. We firstly selected self-assessed health (SAH) status at the onset of the COVID-19 pandemic, which we grouped into four categories (excellent/very good, good, fair and poor). Secondly, we included a dummy variable indicating whether the individual considers his/her health worsened during the pandemic. Thirdly, we incorporated the number of chronic conditions from the following list of diseases: heart attack, high blood pressure, high blood cholesterol, stroke, diabetes, ulcer, Parkinson’s disease, fractures, Alzheimer’s disease, affective disorders, rheumatoid arthritis, osteoarthritis and kidney disease. Since oncological diseases usually require urgent treatments, we disaggregated the information about cancer from the original list of chronic conditions and included it as a specific dummy variable. To capture functional disability prevalence, we added two variables that control for the number of limitations with basic activities of daily living and for the number of limitations regarding instrumental activities of daily living. 

Non-need variables include some characteristics of the household and socio-economic variables. The family status is proxied by a dummy indicating whether the individual lives alone. In addition to the log of equivalent income, we proxied socio-economic status by educational attainment (primary studies, secondary studies and university studies) and labour situation (employed, unemployed, and inactive). Finally, we controlled for the household’s area of residence (urban vs. rural). 

The analyses are performed using Stata SE16© (StataCorp LLC, College Station, TX, USA). Standard errors of HI have been calculated from a bootstrap with 1000 replications. Individual calibrated cross-sectional weights are applied to make the sample representative at country level. Table 1 shows variables’ definitions and summary statistics for the whole sample. Furthermore, Table 2 presents the descriptive statistics of the variables of interest and household income, by country.

## 3. Results

The proportion of people reporting postponed and denied health care reached 25.3% and 5.4% for the whole sample, respectively (Table 1). The share of women in our sample is slightly higher than the share of men (54.6% versus 49.8%), and the average age of the respondents is about 68 years. Most of the adults rate their health as good, very good or excellent (71.3%) and very few report deterioration in their health status during the pandemic (9.3%). On average, individuals suffer from 1.8 chronic conditions, and 3.7% of the whole sample has been diagnosed with cancer. The mean number of limitations affecting basic activities of daily living (0.18) is about a half of those affecting instrumental activities (0.34). The majority of the respondents do not live alone (72.8%), are inactive (57.3%), have secondary level of education (42.1%) and live in urban areas (61.9%).

According to Table 2, half of the countries show percentages of people reporting postponed treatments over the European average, with high differences among them: while the delays are practically inexistent in Bulgaria (1.6%) and very low in Romania (7.2%), they exceed 50% in Luxembourg (53.7%) and Portugal (55.6%). For those countries showing percentages above the average, three different groups may be distinguished: those with percentages about 25–27% (Italy, Spain, Lithuania, Poland and Switzerland), those ranging from 30–35% (Denmark, Slovenia, Belgium, Malta, Czech Republic and France), and those over 50% (Luxembourg and Portugal). Differences in the proportion of individuals reporting denied medical appointments are somewhat lower, ranging from 1.1% (Bulgaria) to 11% (Lithuania). Most countries show percentages below the average, and those with relatively high values belong to three different European areas: south Europe (Portugal and Italy), central Europe (France, Belgium and Luxembourg), and northeast Europe (Poland, Latvia, Estonia and Lithuania).

**Figure 1:**
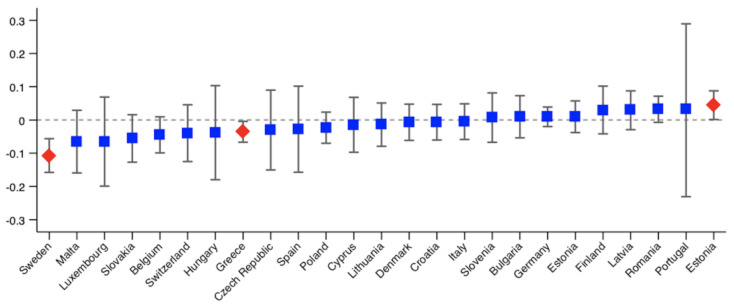
Erreygers’ concentration indices of income-related inequality in postponed health care during the COVID-19 pandemic across European countries. Note: red diamonds indicate *p* < 0.05.

**Figure 2:**
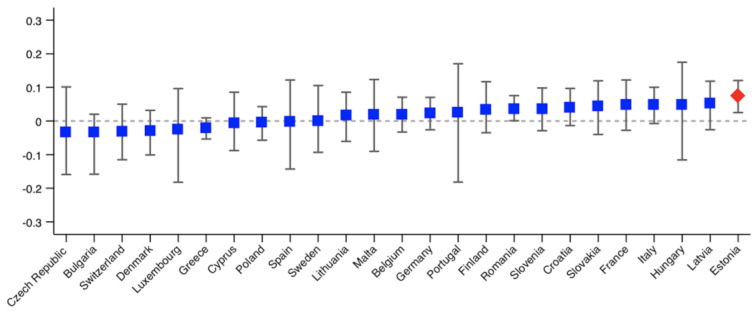
Horizontal inequity indices in postponed health care during the COVID-19 pandemic across European countries. Note: red diamond indicates *p* < 0.05. Bootstrapped bias corrected 95% confidence intervals.

**Figure 3:**
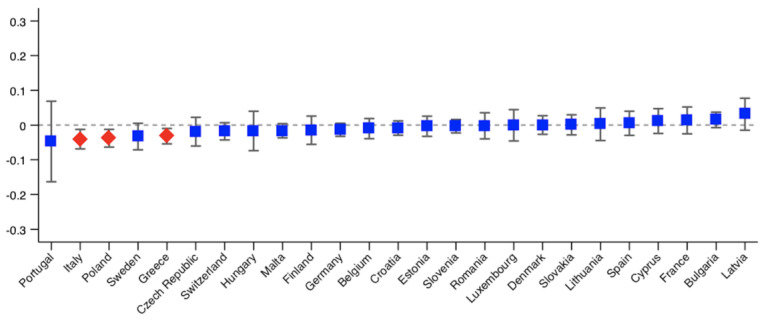
Erreygers’ concentration indices of income-related inequality in denied health care during the COVID-19 pandemic across European countries. Note: red diamonds indicate *p* < 0.05.

**Figure 4:**
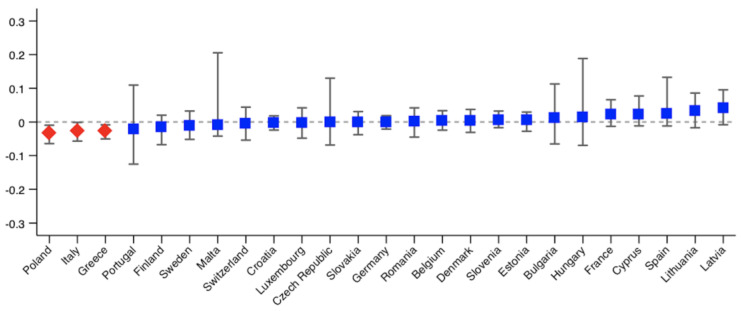
Horizontal inequity indices in denied health care during the COVID-19 pandemic across European countries. Note: red diamonds indicate *p* < 0.05. Bootstrapped bias corrected 95% confidence intervals.

As we analyse 25 countries, we only provide the probit models used to calculate the HI indices for those countries showing significant inequity. The results are shown in the Supplementary Material. The rest of the results are available upon request. Figure 1, Figure 2, Figure 3 and Figure 4 show the Erreygers’ concentration indices and the horizontal inequity indices for the two variables of unmet need, postponed and denied care. These are also detailed in Table 3. Only three countries show significant income-related inequalities in postponed or rescheduled (by a health professional) medical appointments (Figure 1): Sweden, Greece and Estonia, the two former ones favouring the better-off and the latter one favouring the worse-off. When the need component is eliminated from the concentration index, only Estonia shows a significant index, whose sign indicates that pro-poor horizontal inequity exists (Figure 2). However, according to Table 3, two more countries (Italy and Romania) display significant inequity when confidence intervals are calculated at 90% (also favouring the worse-off). Thus, our results reveal that, once need is accounted for, disadvantaged individuals face lower access barriers to health care compared to those belonging to the higher social strata. Regarding denied health care (Figure 3 and Figure 4), three countries show significant pro-rich inequalities and also inequities: Italy, Poland and Greece. Sweden also displays significant inequality with a *p*-value < 0.1, but no inequity emerges in this case after the effect of the need variables is accounted for.

## 4. Discussion

The response to the COVID-19 pandemic across European countries mostly included the postponement of elective treatments and surgeries, as well as non-priority diagnostic tests and medical visits. The proportion of individuals reporting postponed treatments widely varies across Europe, which is partially consistent with the different courses of action taken by different countries. For instance, while Luxembourg and Portugal, reporting percentages of postponed treatments over 50%, cancelled all non-indispensable health care activities since the very beginning of the pandemic (15 and 16 March, respectively), there were no official governmental instructions to postpone non-urgent activity in Bulgaria, which is reflected in the below 2% share of postponed care declared [41]. Ksinan et al. [42] also have shown that postponed medical care in older Europeans was associated, at country level, with more stringent governmental anti-COVID measures.

The percentage of unmet needs due to denied medical appointments is notably lower than that due to postponed care. The between-country variation is also reduced, as it has been observed in previous studies [26,27].

According to our results, only a few countries show significant income-related inequalities in the distribution of postponed health care (Sweden, Greece and Estonia) and denied care (Italy, Greece and Poland). The European countries included in the analyses form a quite heterogeneous group with very different healthcare models (Bismarck in Estonia and Poland, Beveridge in Sweden and Italy and a mixed model in Greece), and high variation in health expenditures and user charges (ranging from significant co-payments in primary and specialised services in Sweden to no user fees in Poland), among other characteristics [43]. Except for Estonia, all the concentration indices suggest inequality favouring the rich. Although it is expected that the reasons for postponing or denying health care in a pandemic context are partially different from those usually considered to compute unmet needs, our results are consistent with the data provided by the European Union about inequality in self-reported unmet needs for medical examinations. In 2019, the percentage of adults reporting unmet needs due to cost, distance or waiting list was systematically higher for the poorest quintile compared to the richest in Sweden, Poland, Italy, and, in particular, in Greece (2.2% vs. 0.5%, 5.6% vs. 3.5%, 4.1% vs. 0.4% and 17.6% vs. 1%, respectively). However, it was lower for the poorest Estonian adults compared to the richest (15.4% vs. 16.5%) [44].

When need is accounted for, again very few countries show significant inequity indices, suggesting that decisions of postponing or denying medical care were mostly related to health care needs. This result is aligned with evidence from the UK suggesting that horizontal equity principle held during the same period for specialised care [25]. As it was pointed out in the introduction, it must be considered that many countries implemented specific measures to reduce barriers to healthcare access, such as extending entitlement to publicly financed care or removing user charges, but also by identifying and helping people most in need or providing income support [3]. Regarding unmet needs due to postponed care, Estonia shows the highest degree of inequity, followed by Italy and Romania. Surprisingly, inequity seems to favour the poor. This is a striking result, particularly for Estonia and Romania, two countries that do not provide universal coverage. Our hypothesis is that severity is not accurately measured through the need variables included in the SHARE, which are those used in the computation of the inequity indices. If, as expected, more severe conditions tend to concentrate on the poorest people, it may be perfectly justified that postponed treatments are more frequently observed among the richest. This hypothesis is consistent with previous evidence showing that the reduction in the use of healthcare services has been higher in patients with mild and less severe conditions, which might suggest that seriously ill patients have been managed properly [5].

Moreover, Italy, Poland and Greece show significant pro-rich inequity in unmet needs due to denied care, which is also consistent with results from previous research. Several studies have identified significant pro-rich inequity in the access to specialist and inpatient care in Italy [35,45,46], not only in the general adult population, but also in older adults [24]. There is also evidence suggesting the existence of significant pro-rich inequity in need-adjusted specialist visits in Poland [47] and Greece [35,45]. More recently, some inequity issues in access to health care have been detected in Greece for vulnerable groups, mostly related to individuals’ insurance status and to important shortages in the supply of health professionals and modern equipment due to the Great Recession [42].

In addition to existing equity problems, the pro-rich inequity found in denied care may be related to difficulties, experienced particularly by vulnerable groups, in getting access to health care in a context where telemedicine gained momentum, especially for the management of chronic diseases that are highly prevalent among the older adults. As Lam et al. [48] have shown for older adults in the United States, telemedicine unreadiness was more common in patients who had less education and lower income. Vergouw et al. [49] also identified non-familiarity with the online eHealth applications as a main barrier to access to and use of eHealth among older adults.

Finally, some limitations should be acknowledged. Firstly, our dependent variables take a value of 1 when medical appointments were postponed/denied by the healthcare centres, and a value of 0 otherwise. Thus, the value of 0 for the variables of interest may indicate two different situations: (a) that the subject did not have any scheduled medical appointment or did not ask for any appointment, or (b) that he/she had scheduled or asked for an appointment, but it was not rescheduled or denied. Unfortunately, the data do not allow to distinguish between the two situations. Also, the estimated models don’t include all potentially relevant information about the individuals, mainly due to a large number of missing values. For example, we do not distinguish between buyers of private health insurance (PHI) policies and those who are only entitled to the public statutory health coverage (only 20.5% of our sample respond to the survey question about health insurance). It is plausible to assume that holding a PHI will significantly influence the probability of reporting rescheduled or denied treatments, since the private sector hardly denies or postpones medical appointments or treatments, not even in times of pandemics. The unavailability of some relevant variables did not allow us to adequately decompose the inequalities in unmet needs. Although we performed the decomposition analysis for those countries with significant concentration indices, we obtained high error terms (the obtained results are available upon request). Additionally, the use of survey data has potential problems related to recall bias. Also, the fact that information is obtained through telephone interviews may affect the response and participation rates, and even the reliability and validity of the answers [50]. Further, since the information of COVID-19 questionnaire was retrieved between June and August 2020, our results only provide a partial picture of unmet health care needs of the European older adults, referred to the first months of the pandemic.

Secondly, we used income from a different wave as the released version of the COVID-19 SHARE questionnaire does not contain updated information on income. We assume that no significant changes occurred in terms of household income from wave 7 to wave 8, since a high proportion of the sample is financially protected by old age pensions (44.4%) or sickness benefits (3.6%). We have used information from wave 7 for all those variables not collected in wave 8 (e.g., area of residence) and we have updated the baseline information collected in wave 7 for the rest of the variables included in the analyses (e.g., chronic conditions or job situation). The same procedure is followed by Smolic et al. [26] or Arnault et al. [27].

Thirdly, linked to the first concern, is the limitation stemming from the methodology. The assessment of horizontal inequity rests on the assumption that once observable need is accounted for, any residual variation in utilization is attributable to non-need factors. As the data available on need indicators are limited, if unobservable variation in need is correlated with income it will result in biased estimates of horizontal inequity [37,51].

However, and despite the above limitations, this study provides useful information about the effects of the response to the COVID-19 pandemic on equity in access to health care for the European older people.

## 5. Conclusions

Our paper contributes to the literature on equity in the utilization of health care and unmet needs among the older people across Europe during the COVID-19 pandemic. Despite the high levels of unmet needs and reorganization (and deprioritization) of care to enable the health system to face the challenges imposed by the pandemic, our findings indicate that inequity in postponed or denied treatments does not seem to be an issue of concern for most European countries, following previous evidence from the United Kingdom [25]. However, our results suggest that Poland, Italy and Greece should pay attention to possible equity problems regarding denied medical care that arose during the first months of the pandemic. Thus, our paper provides new evidence about the consequences of the health crisis management, which is in line with the results from previous studies about horizontal equity in the delivery of health care in the pre-pandemic context for those three countries, as we claimed in the discussion section.

Although important income-related horizontal inequity in unmet needs of European older people during the early waves of the COVID-19 pandemic is not evident for most countries, which is good news, delays in diagnosis and treatment may ultimately translate into adverse health outcomes, reduced quality of life and, even, widen socio-economic health inequalities especially among the older adults, who will represent a growing share of the European population in the next decades. Health policy, therefore, should continue to guarantee equitable access to health care, but should also focus on areas beyond the health sector (education, employment, social protection, etc.) in order to ensure the healthy ageing of the population.

## Figures and Tables

**Table 1:** Descriptive statistics for dependent and independent variables for the whole sample.

Variable	Definition	Mean */Percentage	SD
**Health care**			
Postponed care	Postponed medical appointment since the COVID-19 outbreak (Yes/No)	0.2533	0.4349
Denied appointment	Denied medical appointment since the COVID-19 outbreak (Yes/No)	0.0540	0.2259
**Need variables**			
Female	Gender of respondent (1 female; 0 male)	0.5462	0.4979
Age	Age of respondent (years)	67.75	10.07
	Age groups		
	50–64 years old	0.4728	0.4993
	65–69 years old	0.1363	0.3431
	70–74 years old	0.1323	0.3389
	75–79 years old	0.0964	0.2952
	80–84 years old	0.0890	0.2846
	85 and over years old	0.0732	0.2605
SAH	Respondent’s self-assessed health status		
	Excellent or very good	0.2481	0.4319
	Good	0.4653	0.4988
	Fair	0.2251	0.4177
	Poor	0.0615	0.2402
Worsened health	Respondent’s health status has worsened since the COVID-19 outbreak (Yes/No)	0.0925	0.2898
Chronic	Number of chronic conditions that the respondent has been diagnosed with (excluding cancer)	1.83	1.75
Cancer	Respondent has been ever diagnosed or currently having cancer (Yes/No)	0.0372	0.1894
ADL	Number of limitations with activities of daily living	0.1784	0.7150
IADL	Number of limitations with instrumental activities of daily living	0.3358	1.1702
**Non-need variables**			
Living alone	Respondent living alone (Yes/No)	0.2721	0.4451
Household income	Equivalent household net income (euros)	18,694.16 €	16,886.41 €
Education	Respondent’s highest level of education		
	Primary education	0.3578	0.4794
	Secondary education	0.4211	0.4937
	Higher education	0.2211	0.4150
Current job situation			
	Inactive (retired, permanently sick or homemaker)	0.5729	0.4947
	Employed or self-employed	0.3341	0.4717
	Unemployed	0.0930	0.2905
Urban	Respondent’s living area (1 Urban area; 0 Rural area)	0.6192	0.4856

* Note: weighted means. Due to the small sample size for several countries, the categories of some regressors had to be grouped in a different way to be included in the estimations.

**Table 2:** Prevalence of unmet needs during the COVID-19 outbreak, by country, 2020. (Mean (Std.Dev.)).

Country	Number of Observations	Postponed Care	Denied Care	Equivalent Household Net Income (Euros)
Germany	2446	0.1866 (0.3897)	0.0289 (0.1647)	24,780 (11,978)
Sweden	1107	0.1578 (0.3647)	0.0395 (0.1948)	30,124 (11,566)
Spain	1585	0.2775 (0.4479)	0.0384 (0.1922)	13,249 (7720)
Italy	3008	0.2507 (0.4335)	0.0595 (0.2366)	19,143 (18,864)
France	1772	0.3593 (0.4799)	0.1005 (0.3008)	25,518 (16,502)
Denmark	1823	0.3050 (0.4605)	0.0422 (0.2011)	41,494 (37,022)
Greece	2341	0.1043 (0.3057)	0.0324 (0.1770)	13,056 (17,022)
Switzerland	1637	0.2740 (0.4462)	0.0294 (0.1690)	47,818 (35,662)
Belgium	3297	0.3378 (0.4730)	0.0710 (0.2569)	24,258 (9322)
Czech Republic	2228	0.3720 (0.4834)	0.0332 (0.1792)	8296 (2770)
Poland	2706	0.2775 (0.4479)	0.0682 (0.2522)	6380 (3470)
Luxembourg	719	0.5368 (0.4990)	0.0598 (0.2372)	43,708 (22,753)
Hungary	719	0.2310 (0.4218)	0.0425 (0.2020)	5893 (2449)
Portugal	849	0.5586 (0.4968)	0.0905 (0.2870)	9865 (6030)
Slovenia	2621	0.3128 (0.4637)	0.0300 (0.1705)	10,808 (4870)
Estonia	3514	0.2476 (0.4317)	0.0747 (0.2629)	8296 (4688)
Croatia	1770	0.2195 (0.4140)	0.0293 (0. 1687)	6239 (3442)
Lithuania	1119	0.2757 (0.4471)	0.1100 (0.3130)	5527 (3094)
Bulgaria	773	0.0162 (0.1261)	0.0110 (0.1045)	3215 (1748)
Cyprus	672	0.1839 (0.3877)	0.0419 (0.2004)	32,298 (45,758)
Finland	1192	0.1809 (0.3851)	0.0502 (0.2184)	26,302 (13,524)
Latvia	877	0.1432 (0.3505)	0.0752 (0.2639)	5069 (2707)
Malta	730	0.3495 (0.4771)	0.0219 (0.1466)	11,433 (8215)
Romania	1289	0.0720 (0.2587)	0.0496 (0.2172)	2791 (1693)
Slovakia	812	0.2085 (0.4065)	0.0438 (0.2047)	9631 (8532)
Europe	41,606	0.2533 (0.4349)	0.0540 (0.2259)	18,694 (16,886)

**Table 3:** Income-related inequalities and inequities in postponed and denied care during the COVID-19 pandemic across European countries.

Country	Postponed Care		Denied Care	
	Erreygers CI ^a^	HI ^b^	Erreygers CI ^a^	HI ^b^
Germany	0.0096 (0.0243)	0.0227 (0.0242)	−0.0136 (0.0096)	−0.0007 (0.0099)
Sweden	−0.1085 *** (0.0258)	0.0003 (0.0525)	−0.0325 * (0.0195)	−0.0116 (0.0214)
Spain	−0.0290 (0.0661)	−0.0019 (0.0677)	0.0052 (0.0177)	0.0233 (0.0365)
Italy	−0.0046 (0.0274)	0.0485 * (0.0275)	−0.0409 ** (0.0143)	−0.0277 ** (0.0139)
France	0.0066 (0.0379)	0.0469 (0.0389)	0.0128 (0.0197)	0.0223 (0.0198)
Denmark	−0.0071 (0.0278)	−0.0307 (0.0338)	0.0000 (0.0136)	0.0040 (0.0175)
Greece	−0.0355 ** (0.0160)	−0.0206 (0.0163)	−0.0321 ** (0.0113)	−0.0263 ** (0.0106)
Switzerland	−0.0414 (0.0435)	−0.0311 (0.0425)	−0.0182 (0.0127)	−0.0054 (0.0243)
Belgium	−0.0449 (0.0277)	0.0186 (0.0266)	−0.0099 (0.0148)	0.0034 (0.0153)
Czech Republic	−0.0309 (0.0613)	−0.0333 (0.0673)	−0.0191 (0.0211)	−0.0017 (0.0681)
Poland	−0.0231 (0.0239)	−0.0055 (0.0242)	−0.0381 ** (0.0130)	−0.0343 ** (0.0135)
Luxembourg	−0.0653 (0.0683)	−0.0255 (0.0691)	−0.0006 (0.0229)	−0.0032 (0.0225)
Hungary	−0.0378 (0.0721)	0.0487 (0.0724)	−0.0169 (0.0289)	0.0128 (0.0728)
Portugal	0.0330 (0.1325)	0.0240 (0.0934)	−0.0468 (0.0591)	−0.0221 (0.0595)
Slovenia	0.0093 (0.0324)	0.0353 (0.0313)	−0.0032 (0.0099)	0.0044 (0.0124)
Estonia	0.0445 ** (0.0220)	0.0739 *** (0.0247)	−0.0034 (0.0148)	0.0045 (0.0144)
Croatia	−0.0062 (0.0274)	0.0399 (0.0278)	−0.0088 (0.0106)	−0.0035 (0.0125)
Lithuania	−0.0142 (0.0332)	0.0171 (0.0373)	0.0030 (0.0239)	0.0321 (0.0263)
Bulgaria	0.0094 (0.0150)	−0.0333 (0.0421)	0.0149 (0.0114)	0.0117 (0.0475)
Cyprus	−0.0164 (0.0421)	−0.0066 (0.0437)	0.0112 (0.0182)	0.0229 (0.2060)
Finland	0.0287 (0.0366)	0.0337 (0.0381)	−0.0148 (0.0208)	−0.0155 (0.0210)
Latvia	0.0296 (0.0298)	0.0513 (0.0360)	0.0317 (0.0235)	0.0405 (0.0262)
Malta	−0.0658 (0.0480)	0.0183 (0.0549)	−0.0168 (0.0104)	−0.0097 (0.1025)
Romania	0.0318 (0.0201)	0.0352 * (0.0190)	−0.0024 (0.0192)	0.0013 (0.0218)
Slovakia	−0.0557 (0.0364)	0.0429 (0.0430)	0.0007 (0.0148)	−0.0010 (0.0171)

Note: Sample weights are accounted for. Erreygers’ CI stands for Erreygers’ concentration index, HI stands for horizontal inequity index. Probit models are used to compute need-predicted care. HI is obtained from Erreygers’ CI after controlling for need. ^a^: robust-standard errors in parenthesis. ^b^: boostrapped standard errors in parenthesis. * *p* < 0.1; ** *p* < 0.05; *** *p* < 0.01.

## Data Availability

Restrictions apply to the availability of these data. Data are available at http://www.shareproject.org/data-access.html, accessed on 29 August 2021. This paper used data from SHARE Wave 7 (DOI: 10.6103/SHARE.w7.700) and SHARE COVID-19 Survey (DOI: 10.6103/SHARE.w8ca.100). The SHARE data collection was primarily funded by the European Commission through FP5 (QLK6-CT-2001-00360), FP6 (SHARE-I3: RII-CT-2006-062193, COMPARE: CIT5-CT-2005-028857, SHARELIFE: CIT4-CT-2006-028812), and FP7 (SHARE-PREP: no. 211909, SHARE-LEAP: no. 227822, SHARE M4: no. 261982). Additional funding from the German Ministry of Education and Research, the Max Planck Society for the Advancement of Science, the U.S. National Institute on Aging (U01_AG09740-13S2, P01_AG005842, P01_AG08291, P30_AG12815, R21_AG025169, Y1-AG-4553-01, IAG_BSR06-11, OGHA_04-064, HHSN271201300071C), and from various national funding sources is gratefully acknowledged (see www.share-project.org, accessed on 29 August 2021).

## Supplementary Materials

The following are available online at https://www.mdpi.com/article/10.3390/ijerph18179177/s1, Table S1: Postponed care: marginal effects from the probit model for selected countries. Table S2: Denied care: marginal effects from the probit model for selected countries.
