# Supplementary material for: COVID-19 and Unmet Healthcare Needs of Older People: Did Inequity Arise in Europe?
Source: Int J Environ Res Public Health. 2021 Aug 31;18(17):9177. doi: 10.3390/ijerph18179177 (PMC8431067; doi:10.3390/ijerph18179177)
Supplement: Supplementary file 1 [file ijerph-18-09177-s001.zip › ijerph-1341162-suppl.pdf]

Table S1. Postponed care: marginal effects from the probit model for selected countries.

	Estonia	Italy	Romania
age65_69	0.056754** (0.0283354)	0.0754192** (0.0349337)	-0.0078817 (0.0193587)
age70_74	0.0110328 (0.0306417)	0.0969258** (0.0407151)	-0.0264389 (0.0162161)
age75-79	-0.0133387 (0.0325984)	0.1260776*** (0.0439952)	-0.0029528 (0.0252317)
age80-84	-0.0311275 (0.0331141)	0.0631647 (0.0453217)	-0.0458768** (0.0127484)
age85over	-0.1051319*** (0.0304766)	0.016469 (0.0477944)	-0.0460874* (0.0141844)
female	0.0421899** (0.0194547)	0.0338413 (0.0245423)	-0.0051292 (0.014404)
goodhealth	0.0313858 (0.0450098)	0.0436797 (0.0392839)	0.0114676 (0.0242895)
fairhealth	0.0587566 (0.0452753)	0.1382563*** (0.0473269)	0.0657245* (0.0445912)
poorhealth	0.0678032 (0.0560161)	0.1523483** (0.0698928)	(0.0433943 (0.0436966)
worsened health	0.0736408 (0.0403006)	0.0721135* (0.0425186)	0.021121 (0.024341)
n_chronic	0.032373*** (0.0053468)	0.041235*** (0.007664)	0.010145** (0.0040821)
cancer	0.1002676** (0.0482119)	-0.0346344 (0.0552416)	0.1835515** (0.1085697)
adl	0.0090321 (0.0128977)	-0.0358491** (0.0163343)	0.0243185*** (0.0090289)
iadl	-0.0169343** (0.0085053)	-0.0119525 (0.010183)	-0.016851** (0.0066777)
living_alone	0.0118956 (0.020526)	-0.0054577 (0.0310382)	-0.0054253 (0.0174943)
inactive	0.0069874 (0.0273401)	-0.0635186* (0.0384052)	0.0431548* (0.0193998)
unemployed	-0.0290094 (0.0439907)	0.0325571 (0.0535206)	-0.0109689 (0.0448644)
educmed	0.0513942** (0.0225341)	0.0411084 (0.0307566)	0.0077398 (0.0160744)
educhigh	0.121188*** (0.0291961)	-0.0318934 (0.0405526)	0.0453273 (0.0386935)
ln_income	0.0393017* (0.0215477)	0.0008634 (0.0187911)	0.0229807** (0.0111943)
urban	0.0043146 (0.0193996)	0.0166544 (0.0220703)	-0.0116244 (0.0141759)

* $p<0.1$; ** $p<0.05$; *** $p<0.01$

Table S2. Denied care: marginal effects from the probit model for selected countries.

	Poland	Italy	Greece
**age65_69**	0.0119816 (0.0157648)	0.0318603* (0.019093)	-0.0175009** (0.0069558)
**age70_74**	-0.0159997 (0.013946)	0.0299312 (0.0216315)	-0.0124079 (0.0080501)
**age75-79**	-0.0235822 (0.0147321)	0.0612663*** (0.0268826)	-0.0189956** (0.0069942)
**age80-84**	-0.0089004 (0.0209263)	0.0328357 (0.024751)	-0.0045884 (0.0107624)
**age85over**	-0.0326037 (0.0186079)	0.0157317 (0.0255352)	-0.02159** (0.0064529)
**female**	0.0049614 (0.010306)	0.0194598* (0.0109819)	0.0104301 (0.0069298)
**goodhealth**	0.0325834 (0.0221162)	-0.00095 (0.0185923)	0.0195211 (0.0135518)
**fairhealth**	0.0355674 (0.0304065)	0.0189849 (0.0212631)	0.0101189 (0.0140046)
**poorhealth**	0.0504401 (0.0431913)	0.0374478 (0.0443582)	0.0283928 (0.0279679)
**worsened health**	0.0536666*** (0.0222463)	0.0519768** (0.0269069)	0.0511539*** (0.0221991)
**n_chronic**	0.0111027*** (0.0024113)	0.0065891** (0.0029018)	-0.0001664 (0.0023458)
**cancer**	-0.0063766 (0.022246)	0.0170069 (0.0332322)	0.0381093* (0.0293523)
**adl**	-0.0079012 (0.009288)	-0.0049908 (0.0074602)	-0.0028332 (0.0059536)
**iadl**	-0.0093481 (0.0062185)	-0.0017704 (0.0043554)	0.0051679 (0.0034679)
**living_alone**	-0.0077869 (0.0130446)	0.002927 (0.0124273)	0.0128961 (0.0091062)
**inactive**	-0.0033208 (0.0146049)	-0.0487753*** (0.0195892)	0.0017506 (0.0129643)
**unemployed**	-0.0216527 (0.0166921)	-0.0042639 (0.0224127)	0.0052962 (0.0193454)
**educmed**	0.0136338 (0.0128192)	-0.0197083 (0.0127395)	0.0093903 (0.0092732)
**educhigh**	0.0566152** (0.028868)	-0.0000327 (0.0212258)	-0.003587 (0.0100015)
**ln_income**	-0.0387679*** (0.010712)	-0.0198998** (0.0100884)	-0.0095754 (0.0068784)
**urban**	0.0059477 (0.0108222)	0.0000302 (0.010174)	0.0008576 (0.008614)

* p<0.1; ** p<0.05; *** p<0.01
